# Scaling, growth and cyclicity in biology: a new computational approach

**DOI:** 10.1186/1742-4682-5-5

**Published:** 2008-02-29

**Authors:** Pier Paolo Delsanto, Antonio S Gliozzi, Caterina Guiot

**Affiliations:** 1 Dept. Physics, Politecnico di Torino, Corso Duca degli Abruzzi 24, 10129 Torino, Italy; 2 Dept. Neuroscience, Università di Torino, Corso Raffaello 30, 10125 Torino, Italy

## Abstract

**Background:**

The Phenomenological Universalities approach has been developed by P.P. Delsanto and collaborators during the past 2–3 years. It represents a new tool for the analysis of experimental datasets and cross-fertilization among different fields, from physics/engineering to medicine and social sciences. In fact, it allows similarities to be detected among datasets in totally different fields and acts upon them as a magnifying glass, enabling all the available information to be extracted in a simple way. In nonlinear problems it allows the nonscaling invariance to be retrieved by means of suitable redefined fractal-dimensioned variables.

**Results:**

The main goal of the present contribution is to extend the applicability of the new approach to the study of problems of growth with cyclicity, which are of particular relevance in the fields of biology and medicine.

**Conclusion:**

As an example of its implementation, the method is applied to the analysis of human growth curves. The excellent quality of the results (*R*^2 ^= 0.988) demonstrates the usefulness and reliability of the approach.

## Background

Scaling, growth and cyclicity are basic "properties" of all living organisms and of many other biological systems, such as tumors. The search for scaling laws and universal growth patterns has led G.B. West and collaborators to the discovery of remarkably elegant results, applicable to all living organisms [1-4], and extensible to, e.g., tumors [5-7]. Cyclicity seems to be an almost unavoidable consequence of the feedback of every active biosystem from its environment. In the context of the present contribution we wish to extend the applicability of the Phenomenological Universalities (PUN) approach [8,9], which allows the scaling invariance lost in nonlinear problems to be recovered, to growth phenomena that also involve cyclicities. The latter are particularly relevant in biology and medicine. We wish to make clear from the beginning that only mathematical universalities, as provided e.g. by partial differential equations, represent "true" universalities. As such, in a "top-down" approach, they have been used for centuries. However, we are often challenged, as in the present context, by observational or experimental datasets, from which we wish to "infer" some (more or less) general "laws" using a "bottom-up" approach. PUNs represent a paradigm for performing perform such a task on themost general level.

In muchthe same way that integers are defined as the 'Inbegriff' of a group of objects, when their nature is completely disregarded, PUN's may be defined as the 'Inbegriff' of a given body of phenomenology when the field of application and the nature of the variables involved are completely disregarded. They have been developed [8,9] as a new epistemological tool for discovering, directly from the experimental data, formal similarities in totally different contexts and fields ranging from physics to biology and social sciences. This PUN "classification" can be made conspicuous by means of a simple test based on plots in the plane (*a*,*b*), where *a *and *b *are variables defined in Eq.(1) and (2), respectively.

## Model and methods

In the present context PUN's are formulated as a method f for solving the following problem: given a string of data *y*_*i*_(*t*_*i*_) and assuming that they refer to a phenomenology, which can be reduced to a first order ODE

$$\dot{y}(t)=a(y,t)y(t),$$

we search for a solution *y*(*t*), based not on simple numerical fitting, but on a universal (i.e. absolutely general) framework. The problem, of course, can be generalized to higher order ODE's, PDE's and/or to vectorial, rather than scalar relations, but we prefer here to keep the formalism at its simplest level.

If Eq. (1) refers to growth of any given organism or biological object, *y *is the mass (or length, height, etc.) of the body and *t *is time. To solve such a problem, let us start by assuming that *a *is a function solely of *z *= ln *y *and that its derivative with respect to *z *may be expanded as a set of powers of *a*. It follows that

$$b=\dot{a}=\frac{da}{dz}\dot{z}=\sum_{n=1}^{\infty }\;\alpha_{n}a^{n}(z).$$

If a satisfactory fit of the experimental data is obtained by truncating the set at the *N*-th term (or power of *a*), then we state that the underlying phenomenology belongs to the Universality Class *UN*.

It can be easily shown [8] that the Universality Class *U*1 (i.e. with *N *= 1) represents the well known 'Gompertz' law [10], which has been used for more than a century to study diverse growth phenomena. The class *U*2 includes, besides Gompertz as a special case, most of the commonly used growth models proposed to date in several fields of research, i.e., besides the already mentioned model of West and collaborators [1,4], the exponential, logistic, theta-logistic, potential, von Bertalanffy, etc. models (for a review see Ref. [11]).

Restricting our attention to the class *U*2, by solving the differential equations $\dot{z}=a$ and $\dot{a}=b$, and writing *b*, for brevity as

*b *= *βa *+ *γa*^2 ^

and with the (normalized) initial conditions *y(0) = 1 *and *a(0) = 1*,

$$a=-\frac{\beta }{\gamma }{\left[1-\left(1+\frac{\beta }{\gamma }\right)e^{-\beta t}\right]}^{-1}$$

and

$$y=e^{z}={\left\{1+\frac{\gamma }{\beta }\left[1-exp(\beta t)\right]\right\}}^{\left(-1/\gamma \right)}$$

It is interesting to observe that Eq.(5) can be written as

*u *= *c*_1 _+ *c*_2_*τ*

which shows that the scaling invariance, which was lost due to the nonlinearity of *a*(*z*), may be recovered if the fractal-dimensioned variable *u *= *y*^-*γ*^and the new variable *τ *= exp(*βt*) are considered. In fact, *γ *is, in general, non integer. In Eq. (6), *c*_1 _and *c*_2 _are constants: $c_{2}=-\frac{\gamma }{\beta }$, *c*_1 _= 1 - *c*_2_.

It may also be useful to note that *y *is the solution of the Ordinary Differential Equation (ODE)

$$\dot{y}=\gamma_{1}y^{p}-\gamma_{2}y,$$

where *p *= 1 + *γ *; *γ*_1 _and *γ*_2 _are constants: *γ*_2 _= *β*/*γ *and *γ*_1 _= 1 - *γ*_2_. Their sum is equal to 1, because of the normalization chosen (*y*(0) = 1). Equation (7) coincides with West's universal growth equation, except that here *p *may be totally general, while West and collaborators adopt Kleiber's prescription (*p *= 3/4) [12], which seems to be well supported by animal growth data. For other systems, different choices of *p *may be preferable: in particular C. Guiot et al. suggest a dynamical evolution of p in the transition from an avascular phase to an angiogenetic stage in tumors [13].

Equation (7) may have (as in Refs. [1] to [6]) a very simple energy balance interpretation, with *γ*_1_*y*^*p *^representing the input energy (through a fractal branched network), *γ*_2_*y *the metabolism and $\dot{y}$ the asymptotically vanishing growth. In fact all *UN *(at least up to *N *= 3) fulfil energy conservation (or, equivalently, follow the first Principle of Thermodynamics). However, in *U*1 there is no fractal dimensionality and both input energy and metabolism are proportional to *y*. In *U*2, as we have seen, the energy input term has a fractal dimensionality. In *U*3, one more term with a fractal exponent, again equal to *p*, contributes to growth. In fact, it can be shown (proof omitted here for brevity) that the *U*3 ODE can be written as:

$$\dot{y}+\delta_{1}\frac{d}{dt}(y^{p})=\delta_{2}y^{p}-\delta_{3}y,$$

where *δ*_1_, *δ*_2 _and *δ*_3 _are constants related to the coefficients *β*, *γ*, *δ*, of the truncated U3 series expansion of *b*

*b *= *βa *+ *γa*^2 ^+ *δa*^3 ^

Let us now consider the case

$$a(z,t)=\bar{a}(z)+\tilde{a}(t)$$

in which *a *is assumed to be the sum of two contributions to the growth rate, one ($\bar{a}$), that depends only on *z *(or *y*), while the other ($\tilde{a}$) is solely time-dependent. Then, by writing,

$$y=\bar{y}(t)\tilde{y}(t),$$

it follows

$$\dot{y}=\left(\bar{a}+\tilde{a}\right)\bar{y}\tilde{y},$$

Eq. (1) can consequently be split into a system of two uncoupled equations

$$\dot{\bar{y}}=\bar{a}(z)\bar{y}$$

and

$$\dot{\tilde{y}}=\tilde{a}(t)\tilde{y}$$

Eq. (13) can be solved as before (for the case *a*(*z*)) giving rise to the classes *UN*. A general solution of Eq. (14) can be written as

$$\tilde{a}=\dot{\tilde{z}}=\sum_{n=1}^{\infty }\;A_{n}E_{n}$$

where *E*_*n *_= exp[*i*(*nωt *+ Ψ_*n*_)]. Then, if the sum in Eq.(15) can be truncated to the *M*-th term, we will state that the corresponding phenomenology belongs to the class *UN*/*TM*. It may be interesting to remark that the class *U*0/*TM *and its phenomenology, involving the appearance of hysteretic loops and other effects, has been analyzed in detail, in a completely different context (Slow and Fast Dynamics [9]), under the name of Nonclassical Nonlinearity.

**Figure 1:**
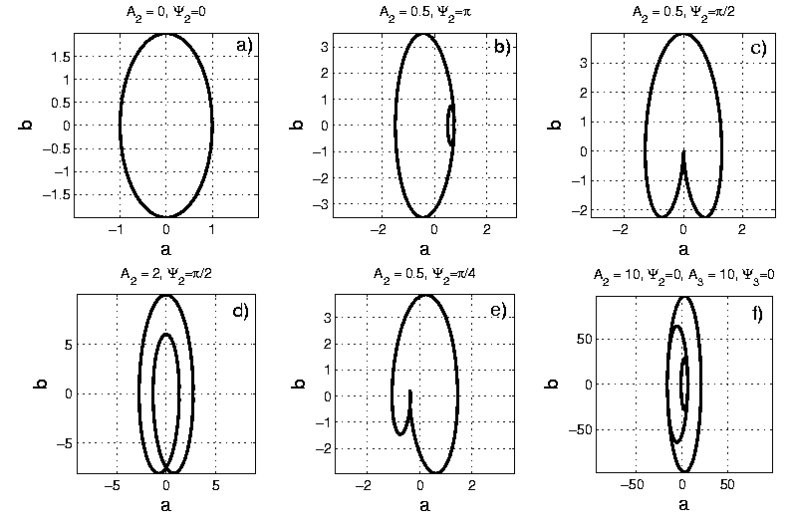
**Examples of *b*(*a*) curves belonging to the class *U*0/*TM***. Examples of *b*(*a*) curves belonging to the class *U*0/*TM*, as described by Eq. (15). We have assumed for all the plots *ω *= 2, *A*_1 _= 1 and Ψ_1 _= 0. As predicted by Eq. (16), in the case *M *= 1 we obtain an ellipse with a ratio *ω *between the two semi-axes. Examples of the *M *= 2 case are shown in the plots (b), (c), (d) and (e), for different choices of the parameters *A*_2 _and Ψ_2_. As expected, three ellipses appear in the case *U*0/*T*3 (plot f).

From Eq.(15) it is easy to obtain $\tilde{z}$, $\tilde{y}$ and $\tilde{b}$ by simple integration or differentiation, but it is no longer possible, from a simple fitting of the experimental curve *b*(*a*), to obtain the relevant UN or TM parameters analytically, keeping, of course, only the real part of Eq.(15). However, assuming that *N *= 0, i.e. $a=\tilde{a}(t)$, (case U0/T1) we can easily see that the curve *b *vs. *a *becomes

$$a^{2}+{\left(\frac{b}{\omega }\right)}^{2}=A^{2},$$

i.e. it represents an ellipse (see Fig. 1a), with *ω *being the ratio between the two semi-axes. In the case *U*0/*T*2, the "interference" between the ellipses generated by the first and second harmonics gives rise to plots, which include two complete ellipses (Fig.1b) or, according to whether *A*_2 _<<*A*_1 _or *A*_2 _> *A*_1_, one complete and one collapsed in a cusp (Fig. 1c) or in a knot (Fig. 1d), respectively. The plot *b *vs. *a *also depends, of course, also on the phase shift between the two harmonics and more complex curves may result (Fig. 1e) if its value is not close to 0 or to *π*. For *N *> 2, the plots obviously become more complex, nevertheless they may often be relatively easy to decipher, as in the *U*0/*T*3 case shown in Fig. 1f.

When *N *≠ 0 the additional problem of interference between $\bar{a}$ and $\tilde{a}$ and between $\bar{b}$ and $\tilde{b}$ arises. However, in the case *UN*/*T*1, if for brevity we write Φ_1 _= *ωt *+ Ψ_1_, from

$$a=\bar{a}+A_{1}cos\Phi_{1}$$

and

$$b=\bar{b}-A_{1}\omega sin\Phi_{1}$$

it follows that

$${\left(a-\bar{a}\right)}^{2}+\frac{{\left(b-\bar{b}\right)}^{2}}{\omega^{2}}=A_{1}^{2},$$

i.e. we still have an ellipse, whose centre, however, moves alongside the $\bar{b}(\bar{a})$ curve. As a consequence, a number (not necessarily integer) *n*_*e *_= Δ*T*/*T *of deformed ellipses is generated (Δ*T *is the time interval considered and *T *= 2*π/ω*). In the case *UN*/*T*0, of course, only one ellipse is visible, since, in the plot *b*(*a*), the ellipse is retraced upon itself any number of times.

**Figure 2:**
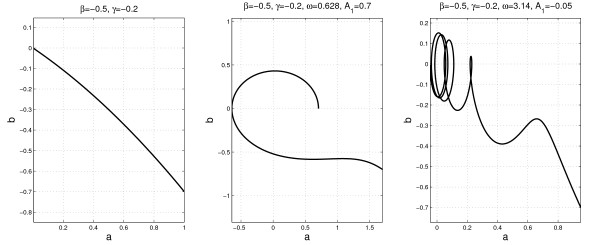
**Interference between U2 and the cyclical term**. Interference between U2 and the cyclical term: (a) *M *= 0, i.e. no cyclical term; (b) *M *= 1 and *n*_*e *_= Δ*T*/*T *= 1, number of periods; (c) *M *= 1 and *n*_*e *_= 5

To illustrate the interference between an U2 curve (Fig. 2a) and the cyclicity contribution, we show in Fig. 2b and Fig. 2c the *b*(*a*) plots in the cases *n*_*e *_= 1 and 5, respectively. In spite of the ellipses' deformation, due to the curvature of the $\bar{b}(\bar{a})$ line, the approximate values of *n*_*e*_, *ω *and *A*_1 _can be retrieved and used as initial values for a fitting of $y=\tilde{y}\bar{y}$, where

$$\tilde{y}=\exp\left(\frac{A_{1}}{\omega }\sin\omega t\right),$$

as it can be immediately obtained from Eq.(14).

In Eq. (20) it has been assumed Ψ_1 _= 0. Such an assumption is justified by the fact that cyclicity is usually due to an interaction of the system being considered with its environement (as a feedback from it), and *t = 0 *is chosen as the time at which the interaction starts.

## Results

As an example of application of the proposed methodology, we consider in the following the curve of human weight development from birth to maturity. We refer to the classical work of Davenport [14] (nowadays an auxological standard), which suggests that the human growth rate exhibits three maxima: one intrauterine, a second one around the 6-th year and a third one other around the 16-th year. The last growth acceleration (adolescent spurt) seems to be activated by the secretions of the pituitary gland and/or the anterior lobe of the hypophysis, while no clear explanations have been proposed for the prenatal and the mid-childhood spurts.

Even if Davenport's finding are still actual, there has been a considerable debate over their interpretation. In fact for man, as for other social mammalians (e.g. elephants, lions, primates), growth development is greatly affected by cyclicity. It has been stated that it cannot be described by a simple curve, but that it requires at least two Gompertz or logistic-like curves (or three for humans [15]), describing the early growth and the juvenile phases separately. The period of extended juvenile growth is most marked in humans, for whom the total period of growth to mature size is very long in comparison with all other mammals.

In addition to the above main accelerations, many authors have observed short-term oscillations in longitudinal data. In the paper of Butler and McKie [16], 135 children were monitored at six monthly intervals from 2 to 18 years of age. Longitudinal studies reveal a cyclic, rhythmic pattern, as a sequence of spurts and lags occurring up to adolescence. In addition, in the paper of Wales [17], very short time cyclicities are reported, such as postural changes in height throughout the day, due to spinal disc compression. Variations in height velocity have also been described with the season of the year, possibly modulated through the higher central nervous system and secretion of melatonin and other hormones with circadian rhythmicity.

**Figure 3:**
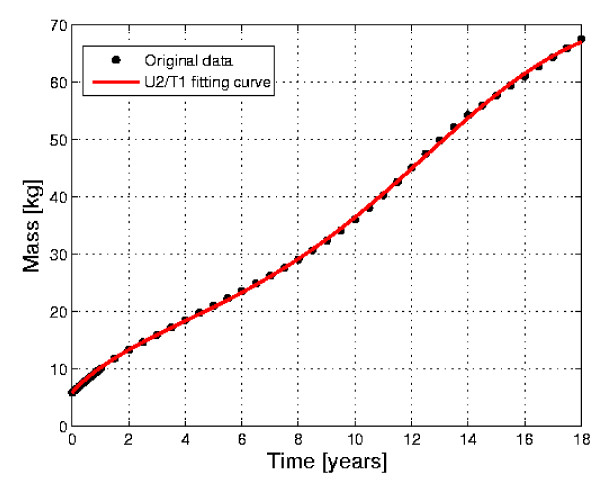
**Curve of human weight development from birth to maturity**. Curve of human weight development from birth to maturity, based on Davenport data (dots) [14]. The solid line represents the fitting obtained with a U2/T1 curve (*R*^2 ^= 0.998).

**Figure 4:**
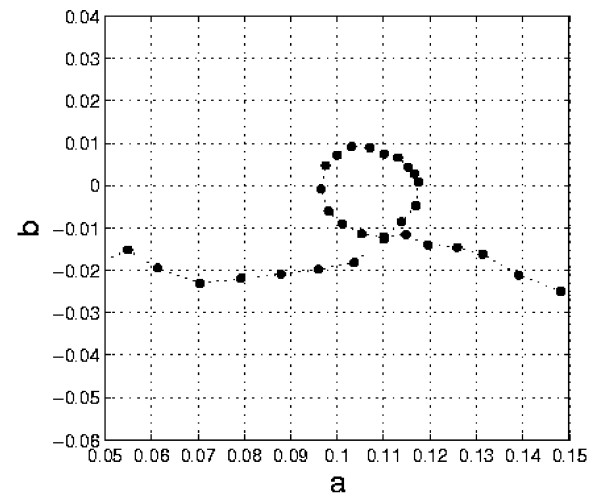
**Evidence of a cyclicity period in the human growth curve**. Evidence of a cyclicity period in the human growth curve (Fig. 3) from the plot *b*(*a*).

**Figure 5:**
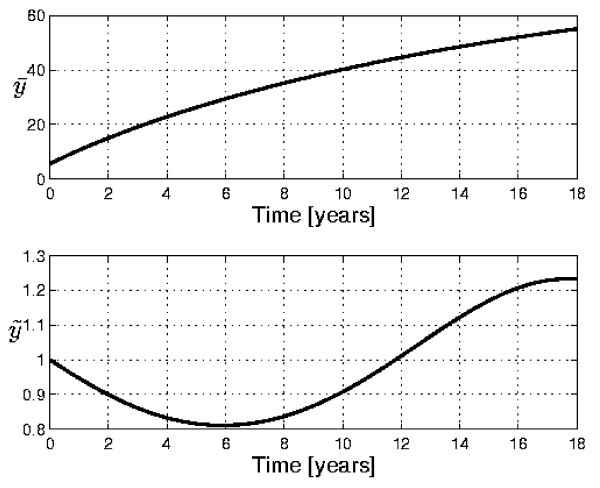
**Separate plots of the curves U2 and T1**. Separate plots of the curves U2 and T1 (see Eqs. (5) and (20)) for the case presented in Figs. 3 and 4. The minimum and maximum of the T1 curve coincide with the times for which the rate of growth is expected to have a minimum or a maximum, in correspondence with the inflection points in the *y*(*t*) curve.

Our method allows us to fit Davenport's curve without the need to consider coupled logistic curves. In Figure 3 we show the original data, relative to human weight development from birth to maturity, and the fitting obtained with the curve U2/T1. The value of *R*^2 ^= 0.998 confirms the correctness of the PUN classification and the accuracy and reliability of the approach. The presence of cyclicity is betrayed by the plot *b*(*a*) in Fig. 4, which clearly exhibits a loop (a very distorted ellipse). Since the curve of Fig. 3 was obtained from 'transversal', instead of 'longitudinal' data, it has been possible to detect only the overall "macroscopic" periodicity. In addition, by separately plotting the curves U2 and T1 vs. time, it is confirmed that the minima and maxima of the T1 curve fall at about 6 years and 17 years, i.e. where the complete U2/T1 curve has its inflection points (see Fig.5).

## Discussion and conclusion

After a short review of the Phenomenological Universalities (PUN) approach, we have proceeded to extend its range of applicability to problems of growth with cyclicity. We have analyzed in detail the case $a(z,t)=\bar{a}(z)+\tilde{a}(t)$, in which the growth rate is assumed to be separable in two terms, depending on *z *= ln *y *and *t *(time), respectively. *y *is the normalized mass (or height, length, etc.) of the body, the development of which is under analysis. As a result, we find that the UN classes, which have been defined and studied for problems without cyclicity, can be generalized as *UN*/*TM *classes, where *TM *represents the solution of the case with only the time dependent term $\tilde{a}(t)$.

In the plots *b *vs. *a *(where *a *and *b *represent the first and second derivatives of *z *= ln *y*, respectively), the presence of cyclicity is betrayed by the appearance of "loops", which look like distorted ellipses, in a number that is equal to Δ*T*/*T*, where Δ*T *is the time range being considered and *T *is the cyclicity period. To be more specific, if we consider, e.g., the class *U*2/*T*1, we have two parameters (*β *and *γ*), which characterize the class *U*2, and two more (*A *and *ω*), related to the cyclcity. From the appearance of the loops it is possible to obtain "initial" or "guess" values of *A *and *ω*, which allow us to fit the experimental or observational data using the *U*2/*T*1 general solution (Eqs. (11), (5) and (20)).

In order to demonstrate the reliability and accuracy of the method, the very important and yet not well understood problem of human growth has been considered. The classical transversal curve of Davenport [15] has been analyzed. The results (Figs. 3 and 5), with a value of *R*^2 ^= 0.998 and the prediction of the acceleration spurts, demonstrate the validity of the approach. More information about human growth mechanisms may be obtained by analyzing longitudinal growth curves for individual or specific groups with the proposed methodology, thus leading to suggestions or evaluations of models incorporating suitable growth mechanisms. Many applications can, of course, be envisaged, such as the diagnosis of undernourishment or diseases, which affect the growth of an individual, or the comparative study of diverse growth patterns in different populations, or the correlation between mass and height development, etc.

An extension of the method presented in this paper to the case of coupled equations, or, more generally, vectorial relations (see e.g. [18]), is in progress.

## Authors' contributions

The first author PPD has developed the general formalism, the second ASG has developed the numerical tools and carried out the numerical analysis and the third CG has suggested and analyzed the applicative context of the paper.
